# Host microsatellite alleles in malaria predisposition?

**DOI:** 10.1186/1475-2875-4-50

**Published:** 2005-10-10

**Authors:** Sonali Gaikwad, Richa Ashma, Nirbhay Kumar, Rajni Trivedi, VK Kashyap

**Affiliations:** 1 Central Forensic Science Laboratory, National DNA Analysis Centre, 30 Gorachand Road, Kolkata-700014, West Bengal, India; 2 Malaria Research Institute, Department of Molecular Microbiology and Immunology, John Hopkins Bloomberg School of Public Health, E5144, 15N.Wolfe Street, Baltimore, MD 21205, USA

## Abstract

**Background:**

Malaria is a serious, sometimes fatal, disease caused by *Plasmodium *infection of human red blood cells. The host-parasite co-evolutionary processes are well understood by the association of coding variations such as G6PD, Duffy blood group receptor, HLA, and beta-globin gene variants with malaria resistance. The profound genetic diversity in host is attributed to polymorphic microsatellites loci. The microsatellite alleles in bacterial species are known to have aided their survival in fatal environmental conditions. The fascinating question is whether microsatellites are genomic cushion in the human genome to combat disease stress and has cause-effect relationships with infections.

**Presentation of the hypothesis:**

It is hypothesized that repeat units or alleles of microsatellites *TH01 *and *D5S818*, located in close proximity to beta-globin gene and immune regulatory region in human play a role in malaria predisposition. Association of alleles at aforesaid microsatellites with malaria infection was analysed. To overrule the false association in unrecognized population stratification, structure analysis and AMOVA were performed among the sampled groups.

**Testing of hypothesis:**

Associations of microsatellite alleles with malaria infection were verified using recombination rate, Chi-square, and powerful likelihood tests. Further investigation of population genetic structure, and AMOVA was done to rule out the confounding effects of population stratification in interpretation of association studies.

**Implication of the hypothesis:**

Lower recombination rate (θ) between microsatellites and genes implicated in host fitness; positive association between alleles -13 (*D5S818*), 9 (*TH01*) and strong susceptibility to *Plasmodium falciparum*; and alleles-12 (*D5S818*) and 6 (*TH01*) rendering resistance to human host were evident. The interesting fact emerging from the study was that while predisposition to malaria was a prehistoric attribute, among *TH01 *alleles; evolution of resistant allele-6 was a recent phenomenon, which could conceivably be driven by infection related selective forces. The host's microsatellite allelic associations with malaria infection were valid in the light of low genetic variance between sampled groups and no population stratification.

## Background

Infectious diseases influence or respond to levels of host genetic variation [1]. As a part of the co-evolutionary process, pathogens had also acquired factors for mitigation and longer survival in the host milieu [2]. *Plasmodium *parasite causes infection of human red blood corpuscles causing malaria. Of its four species, *Plasmodium falciparum *infection is the leading cause of mortality, but the changes in environment and human demography have altered the host-parasite interactions that have subsequently affected the disease spectrum [3]. Malaria provides the paradigm for a disease that has shaped the human genome through natural selection of protective genetic traits. Many molecular studies have documented the associations of human's coding gene polymorphisms such as the haemoglobin variants (Hb E, Hb C, Hb S, α- and β-thalassaemia), G6PD, membrane receptor (Duffy protein), blood group proteins, HLA (HLA-B53, DRB1*1302) and other immune regulatory region with malaria resistance [4,5]. However, human DNA harbours enormous diversity, maintained by neutral polymorphisms such as microsatellites (STR), which are the recombination 'hotspots' owing to repetitive length sequences. It is known that microsatellites present within Opa genes of prokaryotic bacterial species, namely, *Neisseria gonorrhoeae*, *Hemophilus influenzae *aid their survival in fatal environmental conditions [6]. However, it would be fascinating to explore whether high allelic diversity of microsatellite regions in human genome are genomic cushions that has cause-effect relationship aiding species survival against environmental stresses including large number of infections. This paper conjecture is tested and verified at microsatellite loci namely *D5S818 *and *TH01*, present in close proximity to human genes implicated in resistance (reproductive fitness) to malaria.

## Methods

Investigations began after the approval from our Institutional Review Committee and the written consent of patients or the parents of the patients. Blood samples were collected from the patients including clinically positive for *P. falciparum *(*n *= 105) and *Plasmodium vivax *(*n *= 67) infections. The sickle cell anaemic (*n *= 72 represents 49 different families) and β-thalassaemic (*n *= 150 represents 100 different families) patients with no incidence of malarial infection were also sampled. In order to have an overall profile for resistance and susceptibility to malaria, normal healthy individuals (*n *= 174) with no personal history of *Plasmodium *infection and haemoglobin disorder were also studied. The sample collection was restricted to the eastern zone of India.

DNA was isolated from lymphocytes of collected blood specimens following organic extraction protocol [7]. Nested-PCR was used to detect the presence of malaria parasites based on its small subunit ribosomal RNA gene [8]. ARMS-PCR and PCR-RFLP methods were employed to diagnose and confirm the carriers of β-thalassaemia and sickle cell anaemia respectively [9,10]. A total fifteen microsatellite loci including *TH01 *and *D5S818 *were amplified following user's manual and reagents supplied with PowerPlex 16^® ^System (Promega Corporation, Madison, USA). These were subsequently detected on 6% Long Ranger^®^acrylamide gel mounted on ABI 377 DNA Sequencer (Applied Biosystems, Foster City, CA). Genotypes were determined using Genescan™ (version 3.7) and PowerTyper™(version 3.7) software packages.

At first, an admixture model assuming correlated allele frequency between groups for inferring population structure implemented in structure program version 2.0 was employed using genotype data of 13 unlinked markers [11]. Analysis of molecular variance (AMOVA) was carried out on genotype data of unlinked markers between two groups using Arlequin [12]. The proposed hypothesis of this paper was tested by various statistical computations. Recombination rate (θ) as an index of linkage between two loci [13] was computed between the host's *5q31-33 *immune regulatory region controlling *P. falciparum *infection level [14] and *D5S818 *microastellite locus; and between β-globin gene(*11p15.5*) and *TH01 (11p15.5) *microastellite locus. The region from β-globin gene to *TH01 *marker spans 3.83 cM (1 Mb = 1.168 cM). Allele frequencies at each locus in different patients and healthy individuals were estimated by gene count. Likelihood-based tests for allelic associations were performed using the established model [15]. Age of the microsatellite alleles was also calculated [16,17].

## Results and discussion

Microsatellite alleles play a significant role in adaptation of bacteria and perhaps higher organisms to their ever-changing environments. On the downside, they have also been linked to human diseases, production of bile pigment bilirubin and neurotransmitters [6]. Genetic epidemiologists are interested in exploring whether the high allelic diversity in human host has association with large number of infections including malaria or it may be due to population stratification.

**Figure 1:**
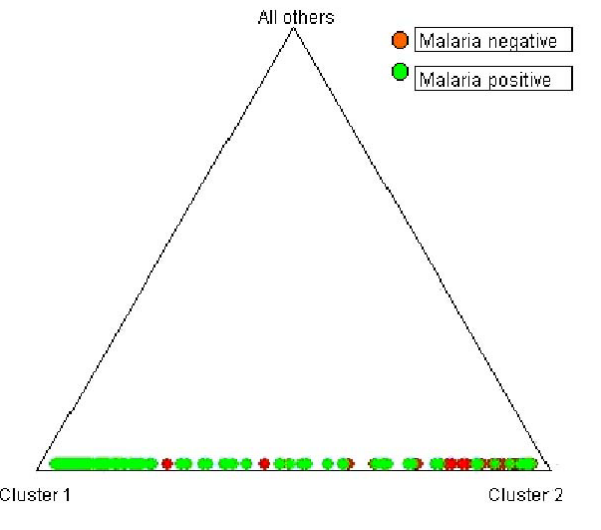
Triangle plot showing estimates of membership coefficient (Q) for each individual by sampled groups, analysed under admixture model, assuming correlated allele frequencies.

**Figure 2:**
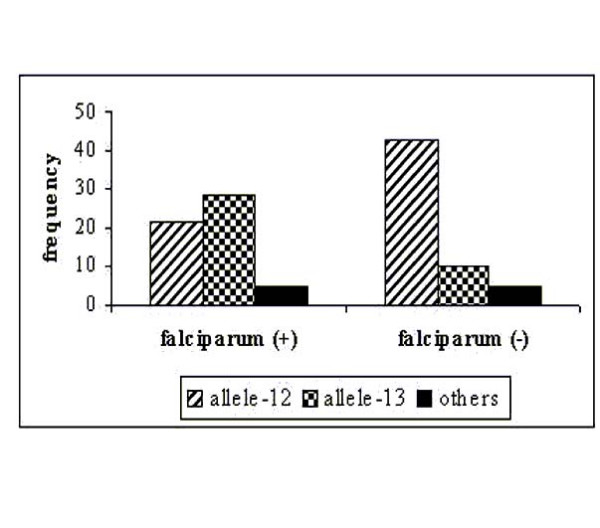
Distribution of allele – 12, 13 and others at microsatellite locus *D5S818 *in positive (+) and negative (-) samples of *P. falciparum *Malaria.

**Figure 3:**
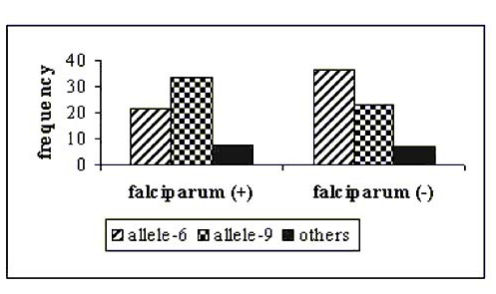
Distribution of allele – 6, 9 and others at microsatellite locus *TH01 *in positive (+) and negative (-) samples of *P. falciparum *malaria.

The evaluation of model-based clustering method showed no evidence of population substructure between sampled groups, which in turn has implication in detecting valid association between a putative candidate allele and malaria disease (Figure 1). Analysis of molecular variance (AMOVA) shows that most of the variance in the sampled population is attributable to within population group variation (98.5%) of the variance and between population group variation is less than 1.5%. The fixation index, F_ST _for the whole sample is only 0.0146. The computed recombination rate (θ) between host's *5q31-33 *and *D5S818 *equals 0.4; and between β-globin gene and *TH01 *was 0.06. These values were lower than 0.5 (cut off) indicating linkage between studied microsatellite markers and genes owing to small genetic distance (cM) as compared to earlier reports [18,19] that clearly depicted significant associations between widely separated loci (17–19 cM) on the same chromosome. The studied tetrameric microsatellite loci manifest varying frequencies of co-dominant alleles in global human populations. Although they are located within blocks of genes subject to selection, alleles numbering 7 to 16 at *D5S818 *and 4 to 9, 9.3, 10–11 and 13.3 at *TH01 *are considered selectively neutral. These alleles differentiated in frequency, age and evolutionary history because on arising they move through population either via genetic drift or being associated with positively selected marker, which may offers evolutionary benefits to the host. Allele frequencies at *D5S818 *locus in *P. falciparum *malaria patients and healthy individuals is presented in Figure 2, which showed the highest occurrence of allele-13 in *P. falciparum *patients and allele-12 in healthy individuals respectively. The distribution pattern in case of *TH01 *(Figure 3) showed allele-9 to be highly frequent in malaria positive isolates while allele-6 was predominant in the normal group with no clinical history of malaria. The Chi-square (χ2) test was applied to check the disproportionate distribution of alleles across two loci in patients and normal groups. Table 1 shows that the estimated values were significantly (P < 0.01) higher than the theoretical table value (9.210), thereby indicating unequal and significant differences in microsatellite allele distribution (frequencies) between *P. falciparum *patients and controls indicate linkage disequilibrium between closely mapped locus and the disease locus. However, weak associations between microsatellite alleles and *P. vivax *infection as well as haemoglobin variants (sickle cell anaemia and β-thalassaemia carriers) were observed. These results might be due to weak and mild malarial infection by *P. vivax *parasite and innate resistance to malaria by haemoglobin disorders. Therefore, the weakly associated samples were excluded from further analysis. Further, statistical analysis employed Likelihood-test, which considered microsatellite loci as bi-allelic markers (associated and non-associated alleles) that are represented in 2 × 2 contingency table. Here, an allele with the higher χ2 value than the table value (at P < 0.01) was taken to be associated. In *P. falciparum *infected group, the likelihood estimate of allele-13 at *D5S818 *was found to be significantly greater than allele-12 (see Table 1), and that of allele-9 at locus *TH01 *was found to be greater than allele-6 (non-associated allele with malaria) indicating a positive association of allele-9 with the disease expression.

**Table 1:** Chi square and Likelihood estimates of alleles – 12, 13 at microsatellite *D5S818*; alleles – 6 and 9 at microsatellite *TH01 *markers (Significance level = 1%) and malaria susceptibility and resistance.

Malaria parasite	Chi-Square values		Likelihood-test estimates	
	
	***D5S818***			
	Normal (allele-12)	Disease (allele-13)	Associated with infection (allele-13)	Non-associated with infection (allele-12)
*P. falciparum*	184.65	109.04	3.56	2.22 × 10^-4^
	***TH01***			
	Normal (allele-6)	Disease (allele-9)	Associated with infection (allele-9)	Non-associated with infection (allele-6)
*P. falciparum*	140.542	128.922	1.75	9.61 × 10^-2^

The prehistoric imprint of host susceptibility to malaria disease was demonstrated by allele-9 (*TH01*) and allele-13 (*D5S818*) because these alleles are estimated to be older (age) than allele – 6 and allele-12. These findings further strengthen the role of host microsatellite alleles in malarial susceptibility as well as disease resistance since people with one version of the gene tend to be resistant to malaria while the other variant make the host susceptible. Furthermore, allele distribution patterns at locus *TH01 *in worldwide populations revealed high frequencies of allele-6 than allele-9 in European populations from Germany, Italy, and Slovakia [20], probably because of zero incidence of fatal *falciparum *malaria and negligible selection pressure. On the other hand, allele-9 in Indian populations [21] suggests primeval imprint of malaria susceptibility and genetic drift. Further, haemoglobin mutations evolved as presumed protective mechanisms against the infection.

## Conclusion

A large number of STR loci dispersed in human genome led to postulation that they act as genomic cushion to combat stress of microbial infections. The study provided significant evidence that an individuals' genotype (microsatellite alleles) is a product of host interaction with *P. falciparum *infection. Thus, positive associations between allele-6 (*TH01*), allele-12 (*D5S818*) and malaria-negative (healthy) individuals demonstrate their role in genetic resistance whereas allele-9 (*TH01*) and allele-13 (*D5S818*) in *P. falciparum *infected individuals illustrate genetic predisposition towards the disease. Furthermore, predominance of allele-9 at *TH01 *(AATG repeat unit) in the individuals inhabiting malaria endemic area suggests genetic predisposition towards malaria as an archaic phenomenon. The host reproductive fitness has evolved during recent times in the form of resistant traits viz., haemoglobin variants (sickle cell mutation, β-thalassaemia) and allele-6 (164 bp) at *TH01 *by deletion of 12 bp from allele-9 (176 bp); both have significantly altered the parasite dynamics. The above findings are further supported by younger ages of microsatellite alleles associated with malaria resistance. A more recent discovery in a low-risk malarial zone shows total absence of allele-9, predominance of lower repeat units at *TH01 *and lack of manifestation of malaria in Jarawa (an aboriginal tribal population of Andaman and Nicobar Island). However, the non-tribal inhabitants (migrants) of this island have higher incidence of malaria, thus substantiating further implication of allele-9 in malaria susceptibility (Kashyap et al. unpublished data). The above findings are valid in the absence of population genetic structure.

## Authors' contributions

VKK is the main investigator who has conceptualized the entire study. RA and SG performed the experiments, collated the data, performed statistical analysis and drafted the manuscript. NK and RT are the co-investigators who reviewed and improved the general presentation of this manuscript. All authors read and approved the final manuscript
